# Editorial: The Potential of Machine-learning in Pharmacogenetics, Pharmacogenomics and Pharmacoepidemiology: Volume II

**DOI:** 10.3389/fphar.2023.1277561

**Published:** 2023-08-29

**Authors:** Augusto Garcia-Agundez, Elena Garcia-Martin, Carsten Eickhoff

**Affiliations:** ^1^ University of California San Francisco, San Francisco, CA, United States; ^2^ University Institute of Molecular Pathology Biomarkers, Universidad de Extremadura, Caceres, Spain; ^3^ Institute for Applied Medical Informatics, University of Tübingen, Tübingen, Germany

**Keywords:** pharmacogenetics, pharmacogenomics, pharmacoepidemiology, machine learning, deep learning

The advent of novel deep learning methods shows great promise in diverse fields of knowledge in pharmacology. Specifically, its capability to quickly process vast amounts of data, discovering complex patterns that would otherwise be imperceptible, will permit crucial advances for drug discovery and repurposing and personalized medicine. In this Frontiers Research Topic, we invited authors to submit their latest research in deep learning applied to pharmacogenetics, pharmacogenomics, and pharmacoepidemiology.

Deep learning methods are valuable tools in discovering the mechanisms of hitherto not fully understood adverse drug reactions. In their study, Akimoto et al. aimed to design a more accurate model to assess drug interactions in drug-induced liver injury (DILI). They found that the combinations diclofenac-famotidine, acetaminophen-ambroxol, and aspirin-cilostazol showed relatively higher excess risk due to a significant interaction. Moreover, both diclofenac and famotidine individually increase the risk of developing DILI. They also found that extreme gradient boosting outperformed more traditional learning algorithms, and was able to reduce overfitting.

Another field where AI can significantly improve clinical pipelines is sifting through vast amounts of inconsistent and sparse data. In Breitenstein et al. study, the authors aimed to identify patterns in medication switches and add-ons in a cohort of epilepsy individuals aged 65 or older by looking at their medication consumption, where despite clinical recommendations being clear, there was lacking evidence on how closely these guidelines were being followed. Their approach was able to capture 92% of all add-ons and 88% of all switches.

In their study, Fang et al. researched the genomic aspects of pathogenesis and drug repurposing in acute type A aortic dissection (ATAAD). Relevant expressed genes were identified, and based on these findings, calcium channel blockers and glucocorticoid receptor agonists were flagged as potentially repurposable drugs to treat ATAAD. An empirical Bayesian test was used to identify the differentially expressed genes through which potential drugs were identified, highlighting the relevance of deep learning techniques in pharmacogenomics for drug repurposing.

Finally, Grant et al. present a study which identifies multi-dimensional *-omics factors relevant to measuring antidepressant response in patients with major depressive disorder with a history of attempted suicide, aiming to discover differential factors that could explain differences in drug effectiveness. In this case, a least squares regression model was employed instead. The authors found the most significant differences between groups in the circadian genes CLOCK and ARNTL.

In this Frontiers Research Topic, we selected four manuscripts for their innovative approach to applying deep learning techniques to vast and multimodal data sources that can be leveraged for pharmacogenetics and pharmacogenomics, particularly in the fields of drug repurposing, -omics and personal factors in drug efficacy, and identifying complex adverse drug events. We are confident that as AI, particularly large autoregressive models, become more efficient, accessible, and adaptable to multiple data types, novel discoveries in these and other fields of pharmacogenetics and pharmacogenomics that were hitherto unattainable will become possible in the coming years. As guest editors for Frontiers in Pharmacology, we are excited to learn what advances scientists across the globe will achieve in pharmacogenetics, pharmacogenomics and pharmacoepidemiology, especially in a context where novel drug research is becoming both more relevant and challenging.

